# *FMR1* Methylation Pattern and Repeat Expansion Screening in a Cohort of Boys with Autism Spectrum Disorders: Correlation of Genetic Findings with Clinical Presentations

**DOI:** 10.3390/genes16080903

**Published:** 2025-07-29

**Authors:** Maria Dobre, Gisela Gaina, Alina Erbescu, Adelina Glangher, Florentina Ionela Linca, Doina Ioana, Emilia Maria Severin, Florina Rad, Mihaela Catrinel Iliescu, Sorina Mihaela Papuc, Mihail Eugen Hinescu, Aurora Arghir, Magdalena Budișteanu

**Affiliations:** 1 Victor Babes National Institute of Pathology, 050096 Bucharest, Romania; maria_dobre70@yahoo.com (M.D.); gisela.gaina@ivb.ro (G.G.); erbescua@gmail.com (A.E.); mhinescu@ivb.ro (M.E.H.); aurora.arghir@ivb.ro (A.A.); magda_efrim@yahoo.com (M.B.); 2 Faculty of Medicine, Carol Davila University of Medicine and Pharmacy, 050474 Bucharest, Romania; emilia.severin@umfcd.ro (E.M.S.); florina2rad@yahoo.com (F.R.); catrinel.iliescu@gmail.com (M.C.I.); 3 Doctoral School, Faculty of Biology, University of Bucharest, 030018 Bucharest, Romania; 4 Prof. Dr. Alexandru Obregia Clinical Hospital of Pathology, 041914 Bucharest, Romania; adelina.glangher@gmail.com (A.G.); linca.florentina@gmail.com (F.I.L.); doinaioana70@gmail.com (D.I.); 5 Faculty of Psychology and Education-Sciences, University of Bucharest, 050663 Bucharest, Romania; 6 Faculty of Medicine, Titu Maiorescu University, 031593 Bucharest, Romania

**Keywords:** Fragile X syndrome, autism, neurodevelopmental conditions, triplet repeat expansion

## Abstract

Background/Objectives: Autism spectrum disorders (ASDs) are neurodevelopmental conditions with early onset of clinical manifestations. ASD etiology is highly heterogeneous, with genetic factors being strong determinants of the behavioral problems and neurodevelopmental deficits. Fragile X syndrome (FXS) (OMIM #300624), caused by the transcriptional silencing of the *FMR1* gene, represents the most common monogenic cause of autism. Our study included 226 boys with a diagnosis of ASD, for a systematic screening of genetic and epigenetic defects in the *FMR1* gene promoter in a Romanian pediatric cohort. Methods: The methods, methylation-specific multiplex ligation-dependent probe amplification (MS-MLPA) and triplet-primed PCR (TP-PCR)/melt curve analysis (MCA), were chosen for their ability to detect the methylation anomalies (the former) as well as repeat expansions in the *FMR1* promoter (the latter). Results: Both methods used in our screening generated concordant results, detecting *FMR1* full mutation in 4 out of 226 patients (~1.8%). This yield is similar to data obtained in larger studies. Three out of four boys presented the typical clinical features, in correlation with genetic findings. Conclusions: The combined use of MS-MLPA and TP-PCR/MCA-based assay was, in our experience, useful to fully describe the genetic defects responsible for FXS. A significant variability of clinical presentations was observed in our small group of children with FXS, from mild to severe intellectual disability and from atypical to characteristic dysmorphic features, as well as various behavioral problems.

## 1. Introduction

Autism spectrum disorders (ASDs) are neurodevelopmental conditions with early onset of clinical manifestations. ASD etiology is highly heterogeneous, involving multiple causes and risk factors, both hereditary and non-hereditary; these factors can act alone or cooperate to generate the behavioral problems and neurodevelopmental deficits [1,2,3,4]. ASD is characterized by impairments in communication and social interaction, sensory anomalies, repetitive, restricted behaviors, and interests [4,5].

Fragile X syndrome (FXS) (OMIM #300624) [6] represents one of the most frequent forms of inherited intellectual disability (ID) and the most common monogenic cause of autism [7,8,9,10]. FXS accounts for ~1% [11] of cases of intellectual disability and 1–2% of cases of autism [12]. FXS is a rare disorder, with a prevalence ranging from 1/3717 to 1/8918 in males of European descent [13,14]. An extensive study of a newborn population of 36,124 males from the USA, including multiple ethnicities, found a point prevalence of 1/5161 [15].

FXS is a condition with X-linked inheritance characterized by variable phenotypic features, which include behavioral and neurologic manifestations, macrocephaly, facial dysmorphic features, and congenital malformations [6].

Fragile X messenger ribonucleoprotein (FMRP) is encoded by the *FMR1* gene, positioned on the Xq27.3 chromosome [7,16]. FMRP has numerous roles, many of which are essential for the development and function of the nervous system. In 99% of the patients, FXS is caused by the expansion to more than 200 repeats of a polymorphic CGG motif, localized in the 5′ untranslated region (5′ UTR) of *FMR1* [17]. This trinucleotide expansion, known as the *FMR1* full mutation, leads to the suppression of *FMR1* transcription by epigenetic mechanisms, mainly hypermethylation. The reduced level or absence of FMRP is considered the main determinant of the clinical features in FXS [18]. In addition, several studies have indicated that copy number variations and sequence variants leading to *FMR1* loss of function may also play a role in the development of FXS [19,20]. Premutations, shorter expansion alleles ranging from 55 to 200 CGG repeats, can also cause different medical problems in some carrier individuals. These are Fragile X-associated primary ovarian insufficiency diagnosed in approximately 20% of female carriers, Fragile X Tremor Ataxia Syndrome detected in older male and female carriers, and Fragile X-associated neuropsychiatric disorder [18,21,22].

In recent years, the advancement of highly sensitive and precise assessment of *FMR1* allele size and methylation status has increased the diagnostic yield. Current guidelines and recommendations acknowledge both the advantages and the limitations of various assays, recommending the use of more than one technique for FXS laboratory diagnostic purposes [21].

In this study, we performed a systematic screening of *FMR1* gene defects, using two different genetic technologies, to evaluate the prevalence of FXS in a cohort of 226 boys with ASD. We report the molecular genetic results of the *FMR1* screening and detail the clinical characteristics of the four individuals with *FMR1* full mutations.

## 2. Materials and Methods

### 2.1. Patient Recruitment and Sample Collection

Our study included 226 boys with a diagnosis of autism spectrum disorder (ASD). All the patients were of Romanian origin and were clinically diagnosed at the Prof. Dr. Alexandru Obregia Psychiatry Hospital (Bucharest, Romania). ASD diagnosis was established using ICD-10 criteria [23] and standardized psychological testing using the Autism Diagnostic Observation Schedule (ADOS) and Autism Diagnostic Interview-Revised (ADI-R). All patients were also assessed by general clinical, neurologic, and dysmorphology evaluation. Ethical approval for the study was obtained from the Ethics Committees of Prof Dr Alexandru Obregia Clinical Hospital of Psychiatry (Protocol approval No. 33/26 November 2019) and Victor Babes National Institute of Pathology (Protocol approval No. 76/3 December 2019).

A written informed consent was signed by all patients’ parents or legal guardians.

### 2.2. Genetic Investigations

Genetic testing was performed at the Victor Babes National Institute of Pathology. The genomic DNA was isolated from whole blood using the PureLink genomic DNA Mini Kit (ThermoFisher Scientific, Waltham, MA, USA) according to the manufacturer’s instructions. The DNA was quantified using a Qubit Fluorometer (ThermoFisher Scientific) and stored at −20 °C until use.

#### 2.2.1. Methylation-Specific Multiplex Ligation-Dependent Probe Amplification (MS-MLPA)

SALSA ME029-B1 FMR1/AFF2 probemix (MRC-Holland, Amsterdam, the Netherlands), with 27 probes that specifically target *FMR1* and *AFF2* genes, was used for MS-MLPA analysis following the manufacturer’s recommendations and a previously described protocol [24,25]. This technique was chosen for the ability to discriminate between normal methylation and hypermethylation and to reveal small deletions or duplications in the targeted genes, with a focus on *FMR1*.

In brief, 100 ng of genomic DNA in 5 μL of TE buffer (10 mM Tris–HCl, pH = 8, and 0.1 mM EDTA) was denatured at 98 °C for 5 min. SALSA MS-MLPA probes (1 fmol each, 1.5 μL total volume) and MLPA buffer (1.5 μL) were added to each sample, followed by incubation at 95 °C for 1 min and hybridization to targets at 60 °C for approximately 16 h. Post-hybridization, 3 μL Ligase buffer and nuclease-free water (NFW) up to a final volume of 20 μL were added to the mixture at room temperature. Subsequently, the mixture was divided into equal volumes in two tubes. At 48 °C, 10 μL of Ligase-65 Master Mix (1.5 μL of Ligase buffer B, 0.25 μL of Ligase-65 and NFW) was added to the first reaction tube (copy number detection); a similar Ligase digestion Master Mix, including methylation-sensitive restriction enzyme HhaI (0.5 μL) (MCR Holland), was added to the second reaction tube (methylation status assessment). Simultaneous ligation and digestion were carried out by incubating the samples at 48 °C for 30 min, followed by heat inactivation of the ligase (at 98 °C, 5 min). The amplification of ligation products was performed in both tubes using Cy5-labeled primers in a PCR reaction with 35 cycles on the Mastercycler Nexus X2 PCR Thermocycler (Eppendorf, Enfield, USA). For each MS-MLPA run, a maximum of 21 patient samples and 3 reference samples from healthy male individuals were used for data normalization. Positive controls for full mutation (DNA samples from an internal control) and non-template controls were also included. Fragment analysis was performed on an ABI 3500 Genetic System using GeneScan™ 600 LIZ as the size standard (ThermoFisher Scientific). Data analysis was conducted using the Coffalyser.Net™ software v.220513.1739.

#### 2.2.2. Triplet-Primed PCR (TP-PCR) and Melt Curve Analysis (MCA)

TP-PCR/MCA is a PCR-based assay that uses primers for unique sequences as well as a primer that anneals to CGG repeats, thus allowing the generation of amplicons from regions with a variable number of short repeats. The results are obtained by analysis of amplicons’ melt peak temperature (Tm).

TP-PCR and MCA were performed according to the protocols reported by Rajan-Babu et al. and Tan et al. [26,27] using a Rotor-Gene 6000 PCR machine (Corbett Research, Qiagen, Hilden, Germany) in a total volume of 25 μL PCR mixture. Each reaction contained 100 ng of genomic DNA, 5 UI HotStarTaq DNA Polymerase (Qiagen, Germany) with 2.5 μL Buffer and 12.5 μL Q Solution, 0.25 μL of dsGreen for Real-Time PCR 10×(Lumiprobe GmbH, Hanover, Germany), dNTP mix (New England BioLabs, Ipswich, MA, USA) with 2 mM final concentration, and a 5:1 ratio (dGTP and dCTP/dATP and dTTP). The sequences of the three used primers were reported by Fu et al., and 0.60 μM of each forward and reverse primer was added, together with a 1000-fold diluted TP-primer [28]. After amplification, PCR products were analyzed by a melt curve generated with Rotor-Gene 6000 series software. Melt peaks were generated by charting the negative first derivative change in fluorescence versus temperature (dF/dT). The Tm was determined as the temperature showing the highest change or decline in fluorescence intensity or the highest dF/dT value.

Five DNA references were purchased from the Coriell Cell Repositories (CCR; Coriell Institute for Medical Research, Camden, NJ, US). These samples were obtained from lymphoblastoid cell lines derived from individuals with different CGG repeat lengths in the promoter region of the *FMR1* gene: NA07174 (30 CGG repeats), CD00014 (56 CGG repeats), NA06892 (86 CGG repeats), NA06891 (118 CGG repeats), and NA07862 (501 CGG repeats).

## 3. Results

### 3.1. Genetic Results

#### 3.1.1. MS-MLPA

**Figure 1:**
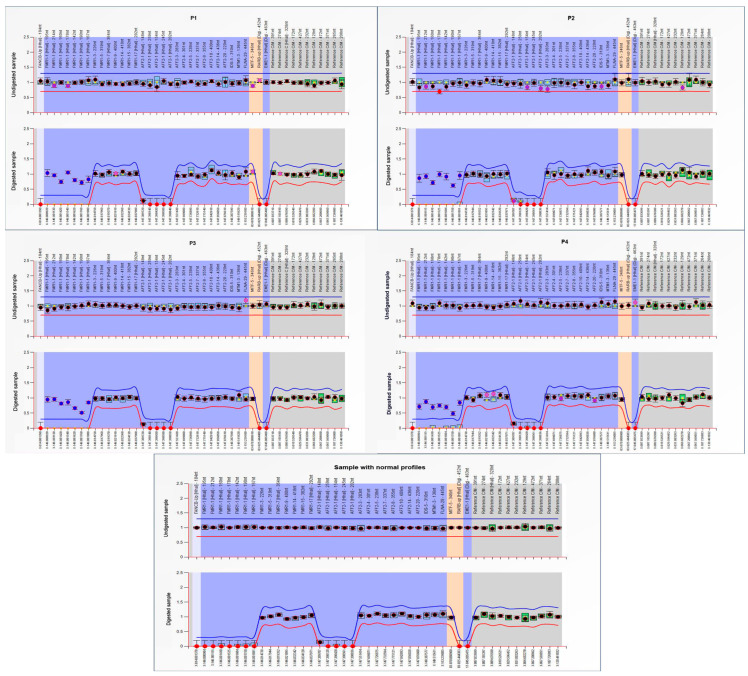
MS-MLPA results for our patients with FXS (P1, P2, P3, and P4 from top-left to bottom-right) showing aberrant hypermethylation pattern of MS-MLPA probes (blue dots in digested sample, lower graph) and no copy number changes (undigested sample, upper graph). The last graph illustrates the normal profiles in both digested and undigested samples (images obtained using Coffalyser.Net™ software, MRC Holland).

For this study, we used the MS-MLPA assay to assess the methylation status of the *FMR1* gene promoter in 226 male patients with ASDs. Four patients from our group presented *FMR1* promoter hypermethylation, known to be generated in the vast majority of patients by a full mutation expansion of trinucleotide repeats. The hypermethylation leads to transcription suppression and consequently to reduced levels of FMRP, which is considered ultimately responsible for the Fragile X syndrome phenotype (Figure 1).

#### 3.1.2. Triplet-Primed PCR

**Figure 2:**
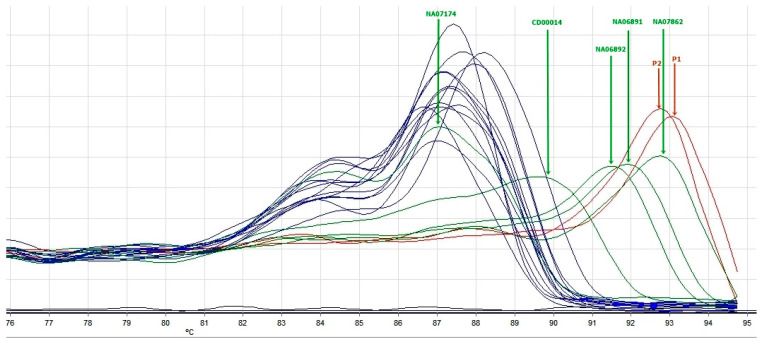
TP-PCR with MCA profiles of *FMR1* testing in male samples. Green peaks correspond to Coriell control samples sized 30, 56, 86, 118, and 501 CGG repeats; blue melt peaks correspond to normal sized samples; red melt peaks were classified as expanded (patients 1 and 2).

**Figure 3:**
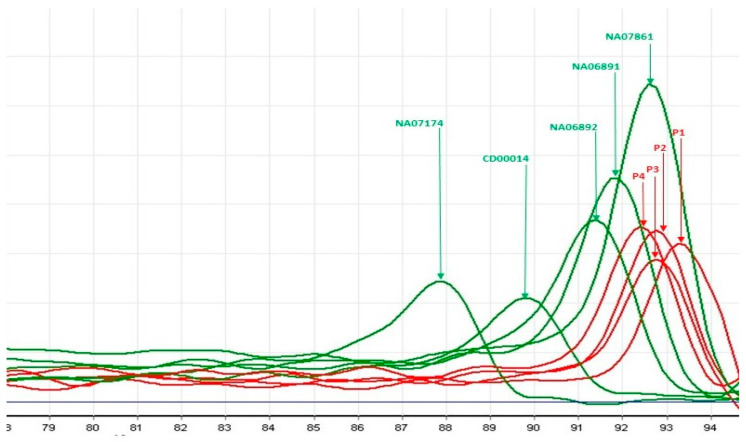
TP-PCR with MCA profiles of the four male patients with ASD found to have an abnormal number of CGG repeats within the range of full mutation (patients 1, 2, 3, and 4, red melt peaks). The green peaks correspond to Coriell control samples sized 30, 56, 86, 118, and 501 CGG repeats.

The screening with TP-PCR and MCA showed that four patients had a Tm, which placed them in the *FMR1* full mutation category. Two patients had a Tm > Tm of NA07862 positive control (501 CGG repeats). The other two had an abnormal number of CGG repetitions with a Tm between that of NA06891 (118 CGG repeats) and NA07862 (501 CGG repeats). The results of TP-PCR were concordant with those obtained by MS-MLPA; the patients with Tm in the range compatible with the full mutation of *FMR1* also presented with hypermethylation of the *FMR1* promoter. The results are reported in Figure 2 and Figure 3.

*FMR1* promoter premutation was detected by TP-PCR in mothers of patients 2, 3, and 4, who were available for testing.

The findings of the MS-MLPA and TP-PCR with MCA investigations provide significant support for the reliability and consistency of both technical approaches. Moreover, the study highlights the utility of the combined use of more than one technique for FXS mutation assessment.

None of these four patients had any copy number variation of potential or known clinical significance detected by chromosomal microarray using the Agilent 4×180k SurePrint G3 Human CGH Microarray Kit (Agilent Technologies, Santa Clara, CA, USA).

### 3.2. Clinical Results

Case 1 is a seven-year-old boy, in maternal assistance since the age of 6 months, who was referred for psychomotor delay; he began walking independently at the age of three and had his first words at 4 years and 6 months. The mother and maternal grandmother were hospitalized in a center for mental diseases, diagnosed both with schizophrenia and severe intellectual disability. The clinical evaluation of the child showed a weight of 20 kg (+2.4 SD), height of 115 cm (+2.9 SD), occipitofrontal circumference of 54 cm (Pc 94), dysmorphic facial features (big, protruding ears, synophris, long eyelashes), open-held mouth, and drooling. Neurological examination showed severe speech delay (he says only four simple words), echolalia, severe intellectual disability (IQ 22), autistic behavior with an ADOS total score of 13, hyperkinesia, and aggressivity. Genetic testing revealed *FMR1* full mutation with abnormal methylation of the *FMR1* promoter (Figure 1) and expansion of CGG repeats (Figure 2 and Figure 3).

Case 2 is a five-year-old boy who was born following a dizygotic twin pregnancy with intrauterine growth restriction. He was delivered by cesarean section at 36 weeks, weighing 2560 g, measuring 49 cm, and with an occipitofrontal circumference of 32 cm. The Apgar score was 7 at 1 min and 8 at 5 min, with good postnatal adaptation. He presented delayed psychomotor development; he sat at 12 months, walked unsupported at 18 months, said the first syllables at 16 months, and first words at 3 years. He had a personal history of gastroesophageal reflux, atopic dermatitis, supraventricular tachycardia, and Wolff–Parkinson–White (WPW) syndrome. The clinical evaluation showed a weight of 18,3 kg (Pc 31), height of 106 cm (Pc 17), occipitofrontal circumference of 52 cm (Pc 73), and dysmorphic facial features (high forehead, strabismus, low-set ears). Neurological examination showed a gait with the tips facing outwards without plantar roll, difficulties going up and down stairs, difficulty with fine and gross motor skills, speech problems (poorly developed language, dyslalia), mild intellectual disability (DQ 68), and autistic behavior (ADOS total score of 10). The EEG showed bilateral central–temporal sharp-wave discharges. *FMR1* full mutation with abnormal methylation of the *FMR1* promoter and expansion of CGG repeats can be visualized in Figure 1, Figure 2 and Figure 3.

Case 3 is a five-year-old boy, born after an uneventful pregnancy at 9 months, with a birth weight of 4300 g, length of 53 cm, and an Apgar score of 9, exhibiting good postnatal adaptation. He had delayed psychomotor development; he walked unsupported at 24 months, said the first syllables at 16 months, and first words at 5 years. The familial history revealed two maternal uncles with intellectual disability. The clinical evaluation showed a weight of 25 kg (Pc 98, +2.1 SD), a height of 115 cm (Pc 91), an occipitofrontal circumference of 53 cm (Pc 92), dysmorphic facial features (big, malformed ears, long nasal philtrum, high palatal arch), and joint hyperlaxity. Neurological evaluation showed a gait with the tips facing outwards, fine and gross motor deficits, hypotonia, speech problems (he says only 3–4 words, uses mostly gestural communication), severe intellectual disability (DQ 22), autistic behavior (ADOS total score of 11), hyperkinesia, and aggressivity. Genetic testing revealed *FMR1* full mutation, namely abnormal methylation and expansion of CGG repeats of the *FMR1* promoter (Figure 1 and Figure 3).

Case 4 is a five-year-old boy, born after an uneventful pregnancy at 39 weeks by cesarian section, with a birth weight of 4050 g, length of 53.5 cm, and an Apgar score of 8 at 1 and 5 min, with good postnatal adaptation. He had a delayed psychomotor development; he sat at 9 months, walked unsupported at 16 months, said the first syllables at 22 months, and first words at 5 years. The personal history showed gastroesophageal reflux and rare respiratory infections. The clinical evaluation showed a weight of 32 kg (>Pc 99, +4 SD), height of 120 cm (Pc 99, +2.4 SD), occipitofrontal circumference of 51 cm (Pc 50), dysmorphic facial features (big, malformed, protruding ears, long eyelashes, strabismus, bilateral epicanthus, broad nasal bridge). Neurological assessment showed a gait with the tips facing outwards, difficulties going up and down stairs, deficits of fine and gross motor skills, hypotonia, speech problems (he says only 3–4 words, use mostly gestural communication), moderate intellectual disability (IQ 46), autistic behavior (ADI-R score 43), hyperkinesia, and aggressivity. The Adaptive Behavior Assessment System testing showed a very low adaptive level (General Adaptive Composite score of 43). Genetic testing revealed *FMR1* full mutation, namely abnormal methylation and expansion of CGG repeats of the *FMR1* promoter (Figure 1 and Figure 3).

## 4. Discussion

In this study, we investigated a cohort of 226 boys with a diagnosis of ASDs for anomalies in the *FMR1* gene promoter using two technical approaches, MS-MLPA and triplet-primed PCR with MCA analysis. From the total number of male patients, four patients (~1.8%) presented hypermethylation of the *FMR1* gene promoter and abnormal number of CGG repeats, consistent with Fragile X syndrome. Thus, the prevalence of FXS in our cohort (1.8%, 4/226) falls within the expected range reported in the literature (1–2.5% of males with ASD) [12].

FXS is one of the most frequent causes of ASDs. In addition to autistic behavior, several other clinical problems can be observed. The typical clinical features of FXS, such as moderate or severe ID, characteristic cranio-facial dysmorphic features, and behavioral problems (hyperkinesia, aggressivity) were present in three out of four boys, in correlation with genetic findings. The fourth patient exhibited autistic behavior and global developmental delay. Speech was more severely affected than motor and cognitive development. Two of the most severely affected children (patients P1 and P3) also had a positive family history for ID and schizophrenia.

Overgrowth was noted in patients P1 and P4, who had weight and height values that deviated by ≥2.4 SD at last presentation. In addition, patient P3 had weight and height values over the 90th percentile. The fourth patient (P2), who had intrauterine growth restriction and normal growth parameters postnatally, was born from a twin pregnancy.

Regarding other medical problems associated with FXS, gastroesophageal reflux was present in two of our cases (P2 and P4); gastroesophageal reflux is one of the most common medical conditions reported in boys with FXS, up to 30% of these patients associating different digestive problems, including gastroesophageal reflux, as a consequence of muscle hypotonia (presented also in our patients) [29]. One patient (P2) presented cardiac anomalies, supraventricular tachycardia, and Wolff–Parkinson–White syndrome. Congenital cardiac conditions represent common features in patients with FXS, including mitral valve prolapse (~50% of cases), aortic root dilatation (25% of cases), and cardiac arrhythmias (24% of patients) [29].

The *FMR1* gene spans 39 kb of genomic DNA and contains 17 exons. The 5′ UTR region of *FMR1* includes a polymorphic CGG tract, between the promoter region and the start codon, with a variable number of triplets [30]. The number of CGG repeats can be stratified into normal (5–44), intermediate (45–54), premutation (55–200), and full mutation length (200+) [21]. CGG tract expansion exceeding 200 repeats leads to epigenetic anomalies, which result in transcriptional silencing of the gene and absence of FMRP [16,31].

The alteration of several mechanisms of epigenetic control, such as DNA methylation, histone modifications, and chromatin remodeling, is considered responsible for *FMR1* gene transcription suppression in FXS [18]. While in individuals with a normal number of triplet repeats, the *FMR1* promoter is protected from methylation by the presence of two flanking regions acting as epigenetic boundaries, the expansion of CGG repeats over 200 (FM) causes DNA methylation to spread beyond these boundaries into the *FMR1* promoter [18]. This abnormal methylation process determines the histone modification’s switch from active to repressive forms, leading to transcriptional block of the *FMR1* [32]. FM CGG repeat expansion also determines the formation of secondary DNA and RNA structures. These secondary structures modify the regulation of chromatin conformation towards the compacted configuration, contributing to *FMR1* silencing [18,32].

FMRP plays crucial roles in brain development and plasticity by selectively binding mRNA molecules and suppressing translation, especially in synaptic compartments [33]. Together with FMRP-interacting proteins, FMRP forms a complex that associates with polyribosomes and represses translation of target mRNAs. Absence of FMRP leads to abnormal dendritic spine morphology and architecture with a major impact on synapse development and function, specifically of excitatory synapses [21,34]. Moreover, FMRP controls the translation of mRNAs encoding K and Ca ion-channels and thus regulates their activity [35].

The molecular diagnosis of FXS has been traditionally based on Southern Blot (SB) analysis, which is a low-throughput and time-consuming technique. Various PCR-based assays were developed for full mutation and premutation detection, advancing the interrogation of FXS defects. The most widely used are TP-PCR assays, with commercial or in-house assays [21]. Methylation-specific assays, such as MS-PCR and MS-MLPA, have been used to detect hypermethylation of the *FMR1* promoter. MS-MLPA assay reliably detects the abnormal methylation profile in male patients with full mutations, without quantifying the number of trinucleotide repeats. MS-MLPA cannot distinguish between a normal number of repetitions or premutation status in male samples or detect any methylation changes in female samples.

Thus, the combined use of techniques that investigate the methylation status, such as MS-MLPA and TP-PCR-based assay capable of assessing the number of CGG repeats in the *FMR1* promoter, is, in our opinion, useful to fully describe the genetic defects in this region responsible for FXS. Our study is, to the best of our knowledge, the first systematic dual-approach investigation of Fragile X syndrome in patients with ASD from Romania.

Our findings highlight once more the importance of including Fragile X testing in the diagnostic algorithm for male patients with ASD, especially those presenting with ID, dysmorphic features, family history of neuropsychiatric conditions, or overgrowth. Early diagnosis of FXS may provide access to tailored interventions and genetic counseling for families. At the same time, our study revealed that Fragile X can be associated with a milder phenotype, even atypical for FXS; thus, a genetic test for this condition should be recommended in all male children with ASD.

One of the strengths of our study is the use of a dual-approach diagnostic strategy combining methylation status and CGG repeat sizing, which improves detection accuracy for FXS. However, limitations include the relatively small number of FXS-positive cases, limiting genotype–phenotype correlations. Additionally, female patients with ASD were not included in this cohort due to study design, although FXS can also occur in females with variable expression.

## 5. Conclusions

The combined use of MS-MLPA and PCR-based assay for *FMR1* promoter repeat expansions and methylation anomalies screening proved successful, with a detection yield of approximately 1.8% (4 out of 226 children). A significant variability of clinical presentations was observed in our small group of children with FXS, from mild intellectual disability and atypical dysmorphic features to severe intellectual disability, characteristic dysmorphic features, and behavioral problems.

## Data Availability

The data generated and analyzed in our study are included in this article.
